# Summary of Keynote Speeches from the 2024 Voice AI Symposium, presented by the Bridge2AI-Voice Consortium

**DOI:** 10.3389/fdgth.2024.1484503

**Published:** 2024-12-03

**Authors:** Rupal Patel, Nicholson Price, Ruth Bahr, Steven Bedrick, Yael Bensoussan, Jean-Christophe Bélisle-Pipon, David Dorr, Christie Jackson, Andrea Krussel, Samantha Salvi Cruz, Jamie Toghranegar, Stephanie Watts, Robin Zhao, The Bridge2AI-Voice Consortium, Yael Bensoussan, Maria Powell

**Affiliations:** ^1^ Department of Communication Sciences and Disorders, Northeastern University, Boston, MA, United States; ^2^ University of Michigan Law School, University of Michigan, Ann Arbor, MI, United States; ^3^ Department of Communication Sciences and Disorders, University of South Florida, Tampa, FL, United States; ^4^ Department of Medical Informatics & Clinical Epidemiology, Oregon Health & Science University, Portland, OR, United States; ^5^ Department of Otolaryngology, University of South Florida, Tampa, FL, United States; ^6^ Faculty of Health Sciences, Simon Fraser University, Burnaby, BC, Canada; ^7^ Office of Health Information and Data Science, Washington University in St. Louis, St. Louis, MO, United States; ^8^ Department of Otolaryngology–Head and Neck Surgery, Vanderbilt University Medical Center, Nashville, TN, United States; ^9^ Department of Otolaryngology, Weill Cornell Medicine, New York, NY, United States

**Keywords:** artificial intelligence, audiomics, voice biomarkers, Bridge2AI, Bridge2AI-Voice

## Abstract

**Introduction:**

The 2024 Voice AI Symposium, hosted by the Bridge2AI-Voice Consortium in Tampa, FL, featured two keynote speeches that addressed the intersection of voice AI, healthcare, ethics, and law. Dr. Rupal Patel and Dr. Nicholson Price provided insights into the advancements, applications, and challenges of AI-driven voice tools in healthcare. The symposium aimed to advance cross-disciplinary collaboration and establish frameworks for the ethical use of AI technologies in healthcare.

**Methods:**

The keynote speeches, delivered on May 1st and 2nd, were 30 minutes each, followed by 10-minutes Q&A sessions. The audio was recorded and transcribed using Whisper (v7.13.1). Content summaries were generated with the aid of ChatGPT (v4.0), and the authors reviewed and edited the final transcripts to ensure accuracy and clarity.

**Results:**

Dr. Rupal Patel’s keynote, “Reflections and New Frontiers in Voice AI”, explored the potential of voice AI for early detection of health conditions, monitoring disease progression, and promoting non-invasive global health management. She highlighted innovative uses beyond traditional applications, such as examining menopause-related symptoms. Dr. Nicholson Price’s keynote, “Governance for Clinical Voice AI”, addressed the regulatory and ethical challenges posed by AI in healthcare. He stressed the need for context-aware systems and dynamic legal frameworks to address liability and accountability.

**Conclusions:**

The 2024 Voice AI Symposium highlighted the transformative potential of voice AI for early detection, health monitoring, and reducing healthcare disparities. It also underscored the importance of dynamic governance to address the ethical and regulatory challenges of deploying AI in clinical settings.

## Reflections and New Frontiers in Voice AI

**Title:** “Reflections and New Frontiers in Voice AI”

**Speaker:** Rupal Patel, MHSc, PhD; Professor of Communication Sciences and Disorders and Computer Science at Northeastern University

Dr. Rupal Patel delivered a compelling keynote speech at the Voice AI Symposium, addressing the significant potential and challenges of voice AI in the healthcare sector. Her talk was titled “Reflections and New Frontiers in Voice AI”, where she underscored the importance of innovative applications in speech and voice health through artificial intelligence. She began by reinforcing the symposium's themes of creating robust data sets, advocating for ethical AI use, raising awareness about voice disorders, and fostering multidisciplinary collaboration.

Dr. Patel elaborated on the use of voice AI to detect early signs of disorders, differentiate between co-occurring diseases, monitor disease progression, and predict future health trajectories. She emphasized the value of voice AI in non-invasive, cost-effective global health monitoring, highlighting its ability to cross linguistic and cultural boundaries. She also discussed potential pitfalls of over-reliance on individual vocal cues such as pitch and loudness, which might vary significantly across cultural and linguistic contexts.

Addressing the practical applications, Dr. Patel noted voice AI's role in diagnosing and monitoring various conditions, including respiratory and cardiovascular disorders, neurological conditions such as Parkinson's disease, and mental health issues like depression and anxiety. She called for more innovative thinking in expanding the use cases of voice AI beyond traditional applications to include under-researched areas such as menopause, which affects a substantial portion of the global female population and exhibits symptoms that overlap with other health conditions.

The speech also touched upon the integration of voice AI with other technologies such as signal processing, natural language processing, wearable computing, and big data analytics. This integration enhances the capability to collect and analyze vast amounts of health data in real time, potentially transforming approaches to health monitoring and diagnosis.

In her closing remarks, Dr. Patel advocated for sparking a global movement to normalize and encourage the widespread use of voice AI in everyday health management, similar to how the ALS Ice Bucket Challenge raised awareness and funds for ALS research. She stressed the importance of ethical considerations, data transparency, and the need to ensure that voice AI technology does not exacerbate existing disparities in healthcare access and quality.

The subsequent Q&A session allowed the audience to engage directly with Dr. Patel, exploring further the themes of de-siloing health data and the practical steps smaller teams can take to contribute to the broader goals of the voice AI field. Dr. Patel's responses highlighted the importance of maintaining a broad perspective while acknowledging the constraints and focused goals of smaller research teams.

Overall, Dr. Rupal Patel's keynote at the Voice AI Symposium provided a thorough overview of the current landscape of voice AI in healthcare, its potential for future growth, and the ethical, cultural, and practical challenges that need to be addressed to fully harness its capabilities.

## Governance for Clinical Voice AI

**Title:** “Governance for Clinical Voice AI”

**Speaker:** Nicholson Price, PhD, JD; Professor of Law at the University of Michigan Law School

In his keynote address, Dr. Nicholson Price provided nuanced insights into the impact of AI on healthcare, emphasizing the urgent need for tailored legal and ethical frameworks to address the unique challenges posed by AI technologies. He critiqued the current regulatory mechanisms, like those of the FDA, which he believes are ill-equipped to manage the differences in AI performance across healthcare settings. Dr. Price advocated for governance that can keep pace with technological advancements and adapt to the variability in AI applications.

A significant portion of his discussion was dedicated to the inherent biases within AI systems. He referenced a study by Obermeyer et al. which demonstrated how an AI system used in healthcare under-assessed the health needs of Black patients by incorrectly using healthcare costs as a proxy for healthcare needs (1). This, Dr. Price noted, is a stark example of how AI can perpetuate, or even exacerbate, existing inequalities unless these systems are rigorously monitored and corrected.

Dr. Price also brought up several instances where AI tools failed when applied in different contexts from where they were developed. He pointed out a case involving a tuberculosis detection AI that incorrectly diagnosed patients based on the type of x-ray machine used, rather than the patients' actual medical conditions, illustrating the need for AI systems to be context-aware and tested across diverse environments (2).

Highlighting the importance of considering human oversight in healthcare AI applications, Dr. Price argued that AI should augment but not replace the nuanced decision-making of healthcare providers when expert providers are available. However, he also stressed the limits of relying on humans in the loop to mitigate risks associated with AI decision-making, given the prevalence of care settings where expert providers are unavailable or overworked.

Throughout his presentation, Dr. Price interwove significant legal considerations, pondering the allocation of liability when AI systems cause harm. He underscored the necessity for developing legal frameworks that account for the shared responsibilities among AI developers, healthcare providers, and regulatory bodies.

During the Q&A session that followed, Dr. Price engaged with the audience on practical ways to implement these frameworks without hampering innovation. He called for continued dialogue among technologists, legal experts, ethicists, and healthcare providers to ensure that AI tools are developed and deployed both ethically and effectively.

In conclusion, Dr. Price's speech offered a thorough examination of the interplay between AI, law, and ethics in healthcare, proposing a balanced approach that maximizes the benefits of AI while diligently addressing its potential risks.

## The Bridge2AI-Voice Consortium

Yael Bensoussan, University of South Florida, Tampa, FL, US; Olivier Elemento, Weill Cornell Medicine, New York, NY, USA; Anais Rameau, Weill Cornell Medicine, New York, NY, USA; Alexandros Sigaras, Weill Cornell Medicine, New York, NY, USA; Satrajit Ghosh, Massachusetts Institute of Technology, Boston, MA, USA; Maria Powell, Vanderbilt University Medical Center, Nashville, TN, USA; Vardit Ravitsky, University of Montreal, Montreal, Quebec, Canada; Jean-Christophe Bélisle-Pipon, Simon Fraser University, Burnaby, BC, Canada; David Dorr, Oregon Health & Science University, Portland, OR, USA; Phillip Payne, Washington University in St. Louis, St. Louis, MO, USA; Alistair Johnson, University of Toronto, Toronto, Ontario, Canada; Ruth Bahr, University of South Florida, Tampa, FL, USA; Donald Bolser, University of Florida, Gainesville, FL, USA; Frank Rudzicz, Dalhousie University, Toronto, ON, Canada; Jordan Lerner Ellis, University of Toronto, Toronto, ON, Canada; Kathy Jenkins, Boston Children's Hospital, Boston, MA, USA; Shaheen Awan, University of Central Florida, Orlando, FL, USA; Micah Boyer, University of South Florida, Tampa, FL, USA; Bill Hersh, Oregon Health & Science University, Portland, OR, USA; Andrea Krussel, Washington University in St. Louis, St. Louis, MO, USA; Steven Bedrick, Oregon Health & Science University, Portland, OR, USA; Toufeeq Ahmed Syed, UT Health, Houston, TX, USA; Jamie Toghranegar, University of South Florida, Tampa, FL, USA; James Anibal, University of South Florida, Tampa, FL, USA; Duncan Sutherland, New York, NY, USA; Enrique Diaz-Ocampo, University of South Florida, Tampa, FL, USA; Elizabeth Silberhoz, University of South Florida, Tampa, FL, USA; John Costello, Boston Children's Hospital, Boston, MA, USA; Alexander Gelbard, Vanderbilt University Medical Center, Nashville, TN, USA; Kimberly Vinson, Vanderbilt University Medical Center, Nashville, TN, USA; Tempestt Neal, University of South Florida, Tampa, FL, USA; Lochana Jayachandran, Mt. Sinai Health, Toronto, ON, Canada; Evan Ng, The Hospital for Sick Children, Toronto, ON, Canada; Selina Casalino, Mt. Sinai Health, Toronto, ON, Canada; Yassmeen Abdel-Aty, University of South Florida, Tampa, FL, USA; Karim Hanna, University of South Florida, Tampa, FL, USA; Theresa Zesiewicz, University of South Florida, Tampa, FL, USA; Elijah Moothedan, Florida Atlantic University, Boca Raton, FL, USA; Emily Evangelista, University of South Florida, Tampa, FL, USA; Samantha Salvi Cruz, Vanderbilt University Medical Center, Nashville, TN, USA; Robin Zhao, Weill Cornell Medicine, New York, NY, USA; Mohamed Ebraheem, University of South Florida, Tampa, FL, USA; Karlee Newberry, University of South Florida, Tampa, FL, USA; Iris De Santiago, University of South Florida, Tampa, FL, USA; Ellie Eiseman, University of South Florida, Tampa, FL, USA; JM Rahman, University of South Florida, Tampa, FL, USA; Stacy Jo, Boston Children's Hospital, Boston, MA, USA; Anna Goldenberg, Hospital for Sick Children, Toronto, ON, Canada.
